# Clinical complications in envenoming by *Apis* honeybee stings: insights into mechanisms, diagnosis, and pharmacological interventions

**DOI:** 10.3389/fimmu.2024.1437413

**Published:** 2024-09-18

**Authors:** Joeliton S. Cavalcante, Pedro Marques Riciopo, Ana Flávia Marques Pereira, Bruna Cristina Jeronimo, Davi Gomes Angstmam, Felipe Carvalhaes Pôssas, Adebal de Andrade Filho, Felipe A. Cerni, Manuela B. Pucca, Rui Seabra Ferreira Junior

**Affiliations:** ^1^ Graduate Program in Tropical Diseases, Botucatu Medical School (FMB), São Paulo State University (UNESP), Botucatu, São Paulo, Brazil; ^2^ Department of Bioprocess and Biotechnology, School of Agriculture, Agronomic Sciences School, São Paulo State University (UNESP), Botucatu, São Paulo, Brazil; ^3^ Center for the Study of Venoms and Venomous Animals of UNESP (CEVAP), São Paulo State University (UNESP), Botucatu, São Paulo, Brazil; ^4^ Minas Gerais Toxicological Information and Assistance Center, João XXIII Hospital, Belo Horizonte, Minas Gerais, Brazil; ^5^ Graduate Program in Tropical Medicine of the State University of Amazonas, Manaus, Amazonas, Brazil; ^6^ Department of Clinical Analysis, School of Pharmaceutical Sciences, São Paulo State University (UNESP), Araraquara, São Paulo, Brazil; ^7^ Center for Translational Science and Development of Biopharmaceuticals FAPESP/CEVAP-UNESP, Botucatu, São Paulo, Brazil

**Keywords:** bee venom, bee sting, clinical envenoming, clinical management, venomous animals, Africanized bee, honeybee

## Abstract

Envenoming resulting from *Apis* honeybee stings pose a neglected public health concern, with clinical complications ranging from mild local reactions to severe systemic manifestations. This review explores the mechanisms underlying envenoming by honeybee sting, discusses diagnostic approaches, and reviews current pharmacological interventions. This section explores the diverse clinical presentations of honeybee envenoming, including allergic and non-allergic reactions, emphasizing the need for accurate diagnosis to guide appropriate medical management. Mechanistic insights into the honeybee venom’s impact on physiological systems, including the immune and cardiovascular systems, are provided to enhance understanding of the complexities of honeybee sting envenoming. Additionally, the article evaluates emerging diagnostic technologies and therapeutic strategies, providing a critical analysis of their potential contributions to improved patient outcomes. This article aims to provide current knowledge for healthcare professionals to effectively manage honeybee sting envenoming, thereby improving patient care and treatment outcomes.

## 1 Introduction

Africanized bees have displayed remarkable adaptability in the Americas representing a great public health concern for humans due to their propensity to attack even in mildly provoked situations. They tend to exhibit a high number of bees that attack from unusually far distances from the hive, persistently pursue their targets for extended periods, and release larger volumes of venom compared to other bee species (1–3). Accidents involving Africanized bees can lead to various clinical manifestations, determined by an individual’s sensitivity to the venom and the number of stings. The most common scenario occurs when an individual not sensitized to the venom receives a few stings. In such cases, the symptoms are typically limited to a localized inflammatory reaction, characterized by redness, pain, and local warmth. Often, these symptoms resolve without the need for medical intervention. Another presentation arises when an individual previously sensitized to one or more components of the venom experiences an immediate Gell and Coombs type I hypersensitivity reaction. This is a severe occurrence that can be triggered by just one sting, necessitating urgent medical attention. Manifestations include swelling of the glottis, bronchospasm, and anaphylactic shock. The third form of presentation results from multiple stings, leading to envenoming with a substantial amount of venom in the body. Alongside local signs such as pain, bleeding, bruising, redness, jaundice, and increased blood flow (hyperemia), these instances are frequently reported (4–8). In addition, systemic manifestations like difficulty breathing (dyspnea), neuroparalytic symptoms, intense burning headaches, nausea, vomiting, weakness (asthenia), muscle and joint pain, severe tremors, kidney failure, rhabdomyolysis, and shock (4–8) can occur due to the diverse fractions of the venom. Incidents leading to these symptoms are not uncommon (2, 9).

## 2 Epidemiology, bee venom, and clinical manifestations

Africanized bees emerged in Brazil during the 1950s when beekeepers introduced African bees (*Apis mellifera scutellata*) to the country. Renowned for their high productivity and disease resistance (10), these bees accidentally escaped and hybridized with European honey bees (*Apis mellifera mellifera*), established in Brazil since the early 19th century. The resulting hybrids demonstrated exceptional adaptability to the tropical climate, rapidly spreading throughout the Americas, excluding Canada (9, 11).

The success of Africanized bees in the Americas is attributed to ecological and genetic advantages over native pollinators, including higher reproductive rates, shorter development cycles, increased drone production, swarming frequency, enhanced disease resistance, and less selective nesting site choice (2, 10). A concerning trend is the escalating incidence of honeybee encounters in urban environments. This increase is primarily linked to pesticide use, deforestation, and declining floral resources, exacerbated by the proximity of bee habitats to human settlements (12). Africanized bee stings occur four to ten times more frequently than those of European bees, often involving group attacks (3, 11). Their extended pursuit of threats and increased venom delivery compared to other bee species (3) pose significant public health risks, leading Brazilian health authorities to classify bee-related incidents as a public health surveillance priority (13).

**Figure 1:**
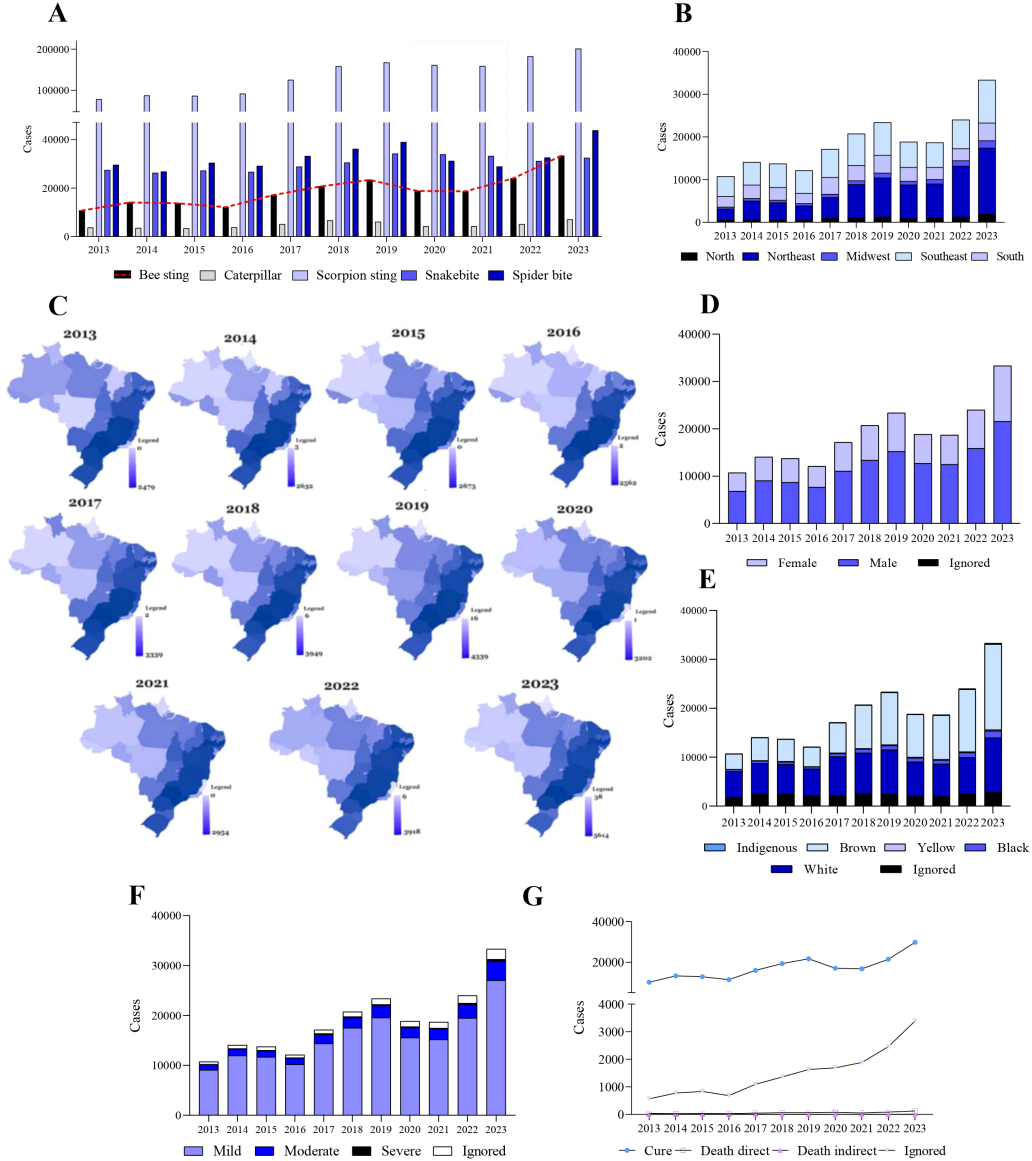
Reported bee accidents and related deaths in Brazil (2013–2023) by state. **(A)** Bee accidents in Brazil per year. Except for 2020 and 2021, the others showed an increasing trend in cases. This drop refers to the years of the pandemic caused by the Sars-Cov-2 virus. **(B)** Annual reported bee accidents related to bee stings per region. **(C)** Distribution of cases of bee accidents according to state. **(D)** Distribution of cases of accidents caused by bees according to sex. **(E)** Distribution of cases of accidents caused by bees according to race. **(F)** Distribution of cases of accidents caused by bees according to clinical classification. **(G)** Distribution of cases of accidents caused by bees according to outcome. Graphs were produced using GraphPad Prism 9.0 software.

In Brazil, between 2013 and 2023, there were 206.746 reported cases of bee stings, with a notable increase in the year 2023, where 33,317 cases were reported, exceeding the number of snake cases (32,420 cases) leading to 649 direct fatalities and an additional 50 deaths indirectly attributed to bee stings (**Figure 1A**). As with envenoming by other venomous animals, the number of cases of envenoming by bees varies between Brazilian states (**Figures 1B, C**), although clinical and epidemiological studies are scarce (7, 14). It is estimated that the lethality rate is about 0.29%, with an annual rate average incidence of 6.89 per 100,000 inhabitants (15), although a temporal increase in reported cases is observed (6, 13, 14, 16, 17). In Amazonas State, the geographical landscape is cited as a contributing factor to the worsening of patients’ conditions (18). This assertion holds merit, considering the geographic obstacles and challenges encountered in route to medical assistance, often leading to fatal outcomes before reaching proper medical care (19). Most reported cases are concentrated in the Northeast, Southeast, and South regions (**Figures 1B, C**), affecting mainly men (**Figure 1D**) people of color (**Figure 1E**). However, the majority of cases are mild (**Figure 1F**) and progress towards cure (**Figure 1G**). However, the impact on patient health in moderate and severe cases is unknown. This is substantiated by the high population density and the diminishing presence of natural vegetation on mountains, in landfills, slums, and urban conglomerates, which can serve as habitats for bees. Consequently, these conditions may provoke bee swarms to launch extensive attacks (20, 21).

**Figure 2:**
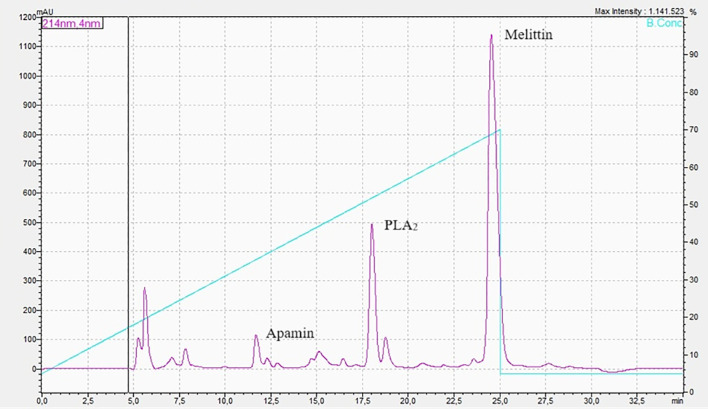
High-performance liquid chromatography of *Apis mellifera* venom.

The *Apis* genus is responsible for most accidents, often resulting in severe outcomes and fatalities (22). Their venom is a complex mixture of proteins, low-molecular-weight peptides, amines, water, and mineral salts. Notably, melittin, phospholipase A_2_ (PLA_2_), hyaluronidase, apamin, and mast cell degranulating peptide are highly toxic components (23, 24). Various analytical methods, including electrophoresis, HPLC, HPLC-MS, GC-MS, liquid scintillation counting, ICP-MS, and stripping voltammetry, have been employed to characterize bee venom (25). Size-exclusion chromatography (SEC-HPLC) under isocratic conditions can identify melittin, apamin, and MCDP. However, due to the melittin/apamine ratio (30:1) and the venom’s chemical complexity, reverse-phase chromatography with C18 columns (RP-18) is necessary for comprehensive analysis, enabling the identification of apamin, hyaluronidase, MCDP, melittin, PLA_2_, procamine, tertiapin, and secapin (26). The abundance of *A. mellifera* venom components is reflected in the intensity of chromatographic peaks, with melittin being the most prominent, followed by PLA_2_, apamin, and other constituents (27) (**Figure 2**).

Immediately following a bee sting, the venom delivery system remains embedded in the skin for approximately 30 seconds, allowing for venom release, with at least 90% injected within the first 20 seconds (28). While removing the stinger is a common initial response, it is unlikely to significantly reduce venom toxicity or quantity absorbed. On average, a bee sting injects 140-150 μg of venom, and the median lethal dose (LD_50_) is 2.8-3.5 mg/kg of body weight (26, 28). Thus, a non-allergic person weighing 60-70 kg could theoretically experience a 50% risk of fatality from 1,000-1,500 stings. However, fatalities have been reported with as few as 200-500 stings. Various factors influence envenoming severity, including time to medical care, age, weight, sting count, and individual characteristics (e.g., immune status, comorbidities, previous sensitization) (29). Individuals with asthma, allergic rhinitis, or a history of bee sting allergies are at increased risk for severe complications (30).

Typically, the clinical manifestations of bee envenomation can be categorized into local inflammatory reactions, allergic reactions, anaphylactic shock, and systemic toxic reactions (3, 9, 29). Initial symptoms are confined to the sting site, often presenting as pain, swelling, redness, and itching. Allergic reactions, classified as type I hypersensitivity reactions, usually occur within 10 minutes of the sting. These reactions can manifest as systemic urticaria, itching, angioedema, vomiting, or diarrhea. In severe cases, allergic reactions may progress to anaphylactic shock, causing bronchoconstriction (26). Importantly, even individuals without allergies can experience anaphylaxis due to systemic mastocytosis.

## 3 Bee venom: molecular basis of physiopathology

A typical bee sting elicits a local inflammatory response characterized by pain, edema, and erythema. Melittin, a primary bee venom allergen, is a potent inducer of acute pain (31). It has been demonstrated that intradermal melittin (5 μg in 50 μL saline) caused severe, 3-minute pain accompanied by local heat and swelling (32–35). Pain intensity and duration correlate with melittin dose, inducing mechanical hyperalgesia at the sting site and heat-thermal hyperalgesia in the surrounding area (34, 35). Animal studies extend these findings to include mechanical and thermal hyperalgesia, edema, and plasma extravasation lasting 72-96 hours (36–39).

Melittin activates the transient receptor potential vanilloid 1 (TRPV1) channel, a nonselective cation channel in peripheral sensory neurons, contributing to pain (40). TRPV1 also mediates pruritus (itching) (41, 42). Histamine, IL-31, IL-4, and cyclooxygenase (COX), lipoxygenase (LOX), and PLA2 pathway products stimulate TRPV1, promoting itching (31, 43). Another mechanism by which melittin induces itching is via serotonin release due to pore formation and mast cell degranulation (31), as well as by increased transcriptional regulation of voltage-gated sodium channels in neurons associated with itch (44, 45). Additionally, melittin enhances nociceptor activity by modulating G protein-coupled receptors (GPCRs), leading to hyperalgesia and allodynia (31).

Another critical effect of venom components, particularly allergens, is hemorrhage. While primarily associated with IgE-mediated anaphylaxis, allergens can also trigger non-IgE-dependent inflammatory responses (46). Bradykinin (BK), whose production increases during envenomation due to melittin’s action, contributes to anaphylactic symptoms. Melittin directly activates PLA_2_, mimicking BK’s effects on tracheal tone and inducing angioedema (47), leading to airway obstruction, asphyxia, and severe gastrointestinal symptoms resembling acute abdomen (46).

A complex interplay between inflammation and coagulation arises, as plasma kinin formation cascade activation results in factor XII binding, autoactivation, and conversion of prekallikrein to kallikrein. Kallikrein cleaves high-molecular-weight kininogen, releasing vasoactive BK (48). BK subsequently activates and modulates coagulation, particularly factor XII (49). Both BK and BK 1-5 inhibit thrombin-induced platelet aggregation, while thrombin plays a crucial role in coagulation and platelet activation (50, 51). Additionally, BK enhances nitric oxide (NO) production in endothelial cells, inhibiting thrombocyte adhesion through angiotensin II blockade (52)

Interestingly, BK induces prolonged thrombolysis via the bradykinin B2 receptor (B2) and prostacyclin (PGI2) (53, 54). This reveals BK’s potential to activate plasminogen, which can be antagonized by angiotensin-converting enzyme (ACE) inhibitors through amplified tissue plasminogen activator effects (53, 55). ACE inhibition modulates fibrinolytic balance, with angiotensin II-mediated increases in plasminogen activation inhibitor-1 playing a crucial role (56). Consequently, coagulation disorders, including decreased fibrinogen activity and prolonged prothrombin and partial thromboplastin times, have been reported in experimental and clinical bee venom envenomation (57).

In summary, melittin induces BK release and ACE dysfunction, leading to coagulation and fibrinolysis imbalances. Additionally, impaired vascular smooth muscle contractility following envenomation exacerbates hemorrhagic episodes (57, 58). Elevated BK levels contribute to reduced vascular tone through epithelial effects (48, 52), while melittin and PLA_2_ may further decrease muscle contractility, potentially inducing hemorrhage.

Bee venom PLA_2_ hydrolyzes phosphatidylcholine, phosphatidylethanolamine, phosphatidylinositol, and phosphatidylserine 2-acyl bonds, disrupting plasma membrane integrity. This process releases lysophospholipids and fatty acids, inducing inflammation and further membrane damage. Notably, purified PLA_2_ lacks hydrolytic activity against erythrocyte membrane phospholipids, but its combination with melittin synergistically enhances hemolysis beyond that achievable by either component alone (59).

Skeletal and cardiac muscle cell membranes exhibit greater resistance to PLA_2_, although both crude venom and melittin exert toxic effects on the cardiovascular system. The exact mechanisms underlying these effects and potential contributions from other venom components remain unclear (60). Melittin initially increases, then decreases the spontaneous beating rate of cultured cardiac myocytes, ultimately causing their degeneration (61).

Wistar rats exhibited electrocardiographic (ECG) changes, enzyme alterations, and morphological lesions resembling acute myocardial infarction (AMI) type 8, suggesting a direct toxic effect of the venom on cardiac muscle, primarily attributed to melittin (62, 63). Bee venom induces extensive endothelial damage, collagen degradation, and smooth muscle cell migration within the aorta (64). Melittin and apamin provoke coronary artery vasospasm, facilitating platelet aggregation and thrombosis. At lower concentrations, melittin induces transient relaxation through an endothelium-dependent mechanism involving NO production and activation of smooth muscle charybdotoxin-sensitive K+ channels, but at higher concentrations, it causes contraction. Apamin, while not directly affecting coronary artery contraction or relaxation, inhibits NO and prostanoid production, modulating the coronary artery’s relaxation response to melittin via smooth muscle apamin-sensitive K+ channels (65). Although bee venom triggers endogenous vasodilatory amine release, paradoxical coronary vasoconstriction remains possible, especially in the context of endothelial damage, potentially leading to acute coronary syndrome (66).

Africanized bee venom induces myonecrosis both *in vivo* and *in vitro* (67). Specifically, melittin triggers phospholipid breakdown, generating free fatty acids and diacylglycerol in equine and human skeletal muscle primary cultures. At higher concentrations, it also breaks down triglycerides. Additionally, melittin alters calcium (Ca^2+^) release thresholds in skeletal muscle terminal cisternae fractions (68). Membrane rupture initiates a cascade of intracellular changes, with increased intracellular calcium levels being a critical factor in subsequent cellular dysfunction and death. Melittin and PLA_2_ synergistically contribute to skeletal muscle cell myonecrosis (69).

*Apis mellifera* venom contains a protein with a conserved C1q domain (70). C1q activates the classical complement pathway by binding to antibody Fc regions, leading to C1r and C1s activation. Subsequently, C1s cleaves C4, generating pro-inflammatory anaphylatoxins C4a and C4b (71). While C1q is present in *A. mellifera* venom, recombinant C1q failed to recognize IgE from bee venom allergic patients (70), suggesting a need for further investigation into its role in complement activation during bee stings.

Interestingly, melittin’s hydrophilic head shares similarities with the C1q sequence. Envenomation induces melittin-mediated, antigen-independent IgG and C1q aggregation, triggering the classical pathway and producing anaphylatoxins C3a and C5a (72). C5a rapidly induces physiological responses, including mast cell degranulation, potentially leading to fatal anaphylaxis (73). Bee sting envenomation elicits acute allergic and inflammatory responses. *A. mellifera* venom and melittin activate the NLRP3 inflammasome, leading to procaspase-1 cleavage and neutrophil recruitment to the sting site. Mast cells play a protective role by degrading and neutralizing *A. mellifera* toxins post-degranulation (74). Melittin activates the 5-lipoxygenase pathway in neutrophils, releasing arachidonate and inducing neutrophilia (75, 76). Additionally, PLA_2_ from *Apis mellifera lamarckii* (Egyptian honeybee) venom hydrolyzes phosphatidylcholine, inhibits platelet aggregation, and impairs blood coagulation by inhibiting the extrinsic pathway (77).

## 4 Role of oxidative stress, inflammation, and coagulation

Redox homeostasis alterations in venomous animal envenomation victims contribute to secondary or long-term complications, with oxidative stress being a key factor. Bee sting envenomation disrupts hepatic metabolism, elevating plasma alanine transaminase (ALT) and aspartate aminotransferase (AST)levels, indicating hepatotoxicity. Additionally, it induces caspase-1 activation and pro-inflammatory molecule secretion through H_2_O_2_ overproduction (78).

One of the most reported clinical complications following bee sting envenomation is ischemic stroke with hemorrhagic transformation. Although the pathophysiology remains unclear, proposed mechanisms involve systemic immune-mediated vasoconstriction and a prothrombotic state, leading to ischemia and subsequent stroke (79), similar to other venomous animal envenomations (80). These events occur through platelet aggregation, coagulation cascade activation via tissue thromboplastin release from damaged tissue phospholipids (81), and rapid declines in platelet count with coagulopathy (82). Combined with hemolysis and endothelial damage, widespread fibrin thrombi formation can lead to vessel occlusion, progressing to hemorrhagic or occlusive transformations.

*Apis mellifera* venom targets various cells, with hemolysis and rhabdomyolysis being particularly damaging due to oxidative stress, primarily mediated by melittin and PLA_2_. Hemolysis can occur directly through venom action or indirectly, while rhabdomyolysis is primarily venom-induced but may also involve inflammation, vascular congestion, and edema (83). PLA_2_-mediated hemolysis results from red blood cell membrane lipid disruption, releasing free hemoglobin (Hb) (83, 84). Free Hb induces macrophage programmed death, shifting macrophage polarization towards a cytotoxic phenotype, exacerbating inflammation and tissue damage (85–87). Additionally, Hb spontaneously oxidizes and reacts with NO, converting hemoglobin to methemoglobin (MtHb).

MtHb is highly pro-oxidant, readily releasing ferric heme, which easily crosses cell membranes and increases oxidative damage. Rhabdomyolysis, another significant contributor to oxidative stress, releases myoglobin, reactive oxygen species (ROS), and uric acid into the bloodstream. Myoglobin undergoes oxidation similar to Hb, releasing free iron and inducing free radical formation. These events can collectively damage the liver and kidneys (83).

Ferroptosis, a ROS-dependent cell death characterized by iron accumulation and lipid peroxidation, may contribute to liver and kidney damage (88). Ferroptosis inducers like erastin or RSL3 increase intracellular iron, generate excessive ROS, and enhance lipid peroxidation through lipoxygenase (ALOX) or EGLN prolyl hydroxylases (PHDs) (88, 89). Hepatocellular death, occurring through apoptosis, necrosis, or pyroptosis, is a common response to various liver diseases (90). Animal studies reveal *A. mellifera* venom-induced sinusoidal and centrilobular congestion, eosinophilia, cytoplasmic vacuolation, intraparenchymal hemorrhage, centrilobular necrosis, and apoptosis (83). Clinically, bee sting envenomation patients exhibit elevated ALT, AST, and bilirubin levels, consistent with hemolysis, thrombotic microangiopathy, and acute liver injury (91). Hepatic vessel occlusion due to microthrombi formation can also lead to ischemia and necrosis (92). Additionally, venom-induced oxidative stress, evidenced by increased malondialdehyde (MDA) and glutathione (GSH) levels, contributes to liver damage (93).

Elevated MDA and GSH levels indicate lipid peroxidation in liver tissue, a process mediated by ROS generated through various mechanisms, including neutrophil activity (94). This lipid peroxidation serves as a pivot for inflammation, as evidenced by increased TNF-α levels in liver tissue (93). The potential involvement of neutrophil extracellular traps (NETs) in this process warrants further investigation. Nox enzyme-dependent NET formation is well-characterized, involving increased ROS production, neutrophil granule and nuclear membrane disintegration, and the release of neutrophil elastase (NE) and myeloperoxidase (MPO) (95). NE and MPO interact with the neutrophil nucleus, cleaving histones and facilitating chromatin decondensation, ultimately leading to the release of DNA decorated with granular content into the extracellular environment for antigen capture (96, 97). While NET formation has been observed in snakebite envenomation (98), its role in multiple bee sting envenomation remains unexplored.

Oxidative stress induced by bee venom contributes to acute kidney injury. Bee venom and melittin disrupt renal cell redox homeostasis by inhibiting α-MG uptake through a PLA2-oxidative stress-Ca2+ pathway (99). This process involves increased arachidonic acid and lipid peroxide production, along with elevated Ca2+ uptake, suggesting a link between PLA_2_ activation, Ca2+, and oxidative stress in renal cells (99). Acute kidney injury, primarily affecting proximal tubules, is a common complication of bee sting envenomation (100, 101). Redox imbalance, characterized by lipid peroxidation and membrane protein denaturation, leading to altered membrane fluidity, enzyme function, and ion transport, plays a crucial role in renal failure pathogenesis. The kidney’s high sensitivity to oxidative stress due to its rich polyunsaturated fatty acid content renders it susceptible to tubular necrosis caused by bee venom-induced redox imbalance (102).

Disseminated intravascular coagulation (DIC) with associated thrombocytopenia presents another potential mechanism for kidney injury. Despite its prevalence, effective treatment and management strategies remain limited. Given platelets’ sensitivity to external stimuli, including ROS, their interplay with oxidative stress is plausible, especially considering platelet apoptosis exacerbates oxidative stress induced by the hemorrhagic, hemolytic, and necrotic effects of bee venom components. Numerous case studies have documented initial thrombocytopenia and alterations in hemostatic and renal systems. Platelets play a crucial role in thromboinflammation (80), explaining the pathogenesis of renal changes in bee sting envenomation.

Roodt et al. (83) reported pulmonary congestion, septal enlargement, atelectasis, emphysema, intra-alveolar hemorrhagic foci, and arterial lesions with acute edema in mice following experimental envenomation with *Apis mellifera mellifera* venom from different regions of Buenos Aires, Argentina. While pulmonary alterations in bee envenomation remain unclear clinically, proposed mechanisms include pro-thrombotic state-induced congestion due to platelet aggregates, fibrin deposition, and erythrocyte accumulation, similar to snake envenomation. Pulmonary edema may result from catecholamine-induced myocarditis, myocardial ischemia due to coronary vasoconstriction, and direct cardiotoxin effects on the myocardium (103–106). Additionally, blood leukocyte mobilization, as observed in other venomous animal envenomations, could contribute to acute lung injury.

## 5 Clinical complications in honeybee stings envenoming

**Figure 3:**
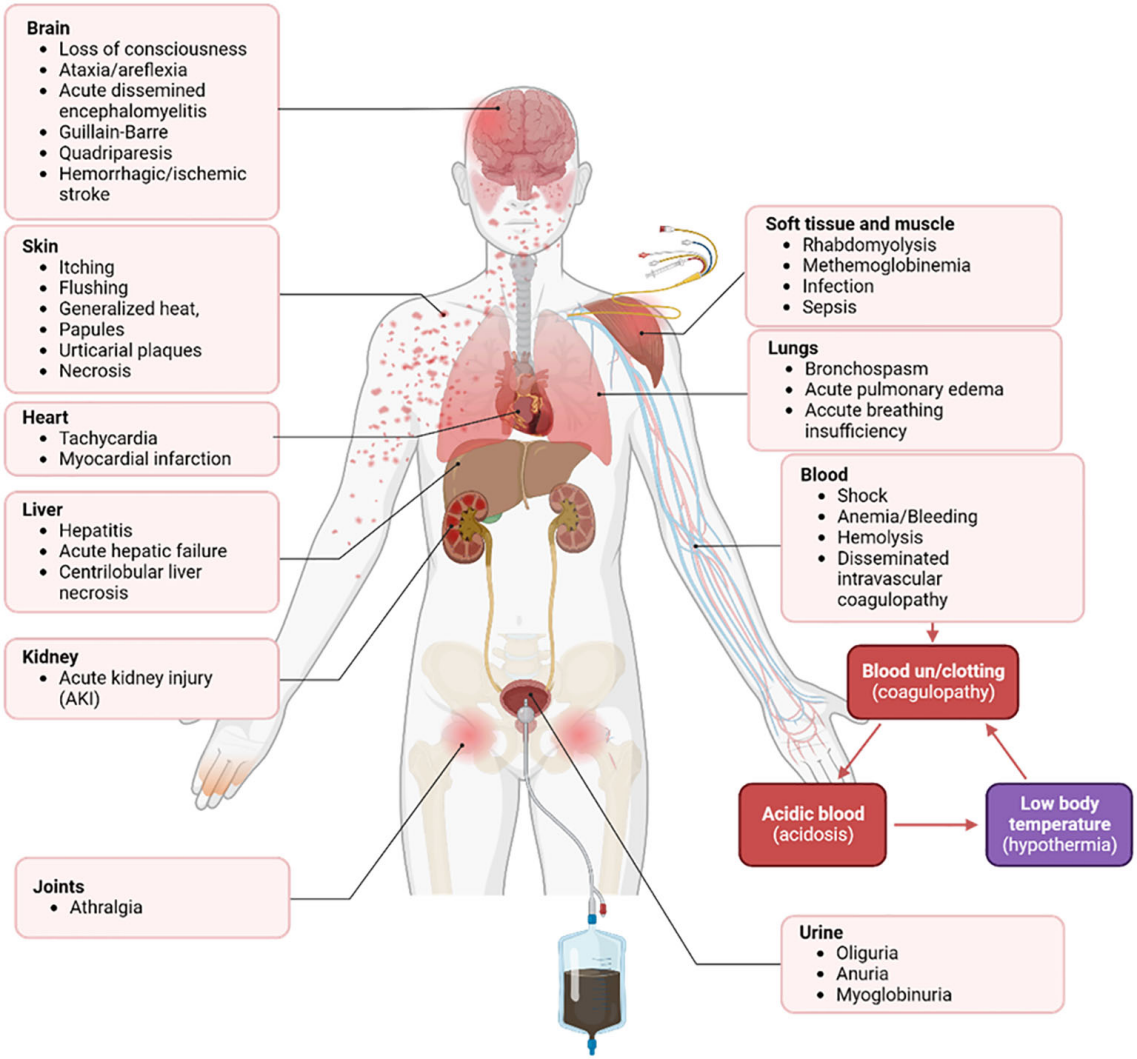
Spectrum of severe systemic effects from bee stings. Multiple bee stings trigger highly severe systemic reactions, exhibiting a broad spectrum of clinical manifestations.

A wide array of clinical complications can arise from multiple bee stings (**Figure 3**). These complications manifest locally or systemically, with variable onset, organ involvement, and overall impact. While frequently reported, the underlying pathogenesis of rarer reactions remains largely undefined (**Table 1**).

**Table 1:** Complications associated with honeybee stings envenoming.

System/Organ	Clinical complications	Reference
**Cardiovascular**	Acute myocardial ischemia	(107–109)
	Acute myocardial injury	(110)
	Atrial fibrillation	(111)
	Hypertension	(112, 113)
	Kounis syndrome (KS)	(111, 114–120)
	Left ventricular hypertrophy	(121)
	Left ventricular systolic dysfunction	(108, 122)
	Mobitz type 2 heart block	(123)
	Myocardial damage	(112, 124)
	Pericardial effusion	(125)
	Pericarditis epistenocardica	(126)
	Subendocardial hemorrhage	(121)
	Takotsubo cardiomyopathy	(127)
**Digestory**	Boerhaave’s syndrome	(107)
	Gastrointestinal hemorrhage	(113, 128)
**Hematologic**	Acute limb ischemia	(129)
	Acute femoral thrombosis	(129)
	Anemia	(130)
	Bleeding	(4)
	Brachial artery thrombosis	(131)
	Deep vein thrombosis (DVT)	(132)
	Disseminated intravascular coagulation	(112)
	Hematochezia	(133)
	Hemolysis	(29, 91, 112, 134)
	Shock	(18, 112, 125, 135, 136)
	Thrombotic microangiopathy	(91)
	Thrombotic thrombocytopenic purpura (TTP)	(137)
**Hepatic**	Acute liver injury	(91)
	Adrenal hemorrhage	(138)
	Hepatic damage	(124)
	Hepatic dysfunction	(29, 112)
	Ischemic hepatitis	(133)
**Immune**	Anaphylactic shock	(4, 73, 107, 108, 114, 121, 122, 133, 139–143)
	Mast cell activation syndrome	(116)
**Multisystem**	Cardiopulmonary arrest	(143)
	Multiorgan failure	(79, 92, 134, 136, 144–146)
	Multiorgan injury	(147)
**Muscular**	Hemiparesis	(148, 149)
	Rhabdomyolysis	(18, 29, 112, 135, 148, 150–155)
**Nervous**	Acute bilateral cerebellar infarction	(156)
	Axonal motor polyneuropathy	(157, 158)
	Cavernous sinus thrombosis	(159)
	Coma	(112, 136)
	Convulsion	(159)
	Encephalitis	(128)
	Guillain-Barre syndrome	(160)
	Hemorrhagic/Ischemic stroke	(134, 136, 148, 149, 161–165)
	Intracerebral hemorrhage	(136)
	Multiple acute cerebral infarcts	(148, 149, 159)
	Subarachnoid hemorrhage	(4, 121, 149)
	Subdural hemorrhage	(149)
	Tonsillar herniation	(133)
	Transcortical motor aphasia	(166)
**Renal**	Acute kidney injury	(29, 91, 108, 113, 135, 150, 153, 155, 159, 167)
	Acute kidney failure	(18, 112, 134, 136, 147, 148, 151, 154)
**Respiratory**	Acute pulmonary emphysema	(141)
	Acute respiratory distress syndrome (ARDS)	(18, 112, 115, 125, 134, 135, 168)
	Bronchial obstruction	(141)
	Laryngeal congestion	(138)
	Laryngeal edema	(138, 139)
	Pulmonary congestion	(139, 141, 169)
	Pulmonary edema	(113, 125, 127, 139, 141)
	Pulmonary hemorrhage	(141)
	Traquea congestion	(138)
	Traquea edema	(138)
**Skin**	Angioedema	(169, 170)
	Grover’s Disease (GD)	(171)
**Urinary**	Anuria	(115, 125)
	Hematuria	(130, 133)
	Hemoglobinuria	(29)
**Visual**	Cataract	(172, 173)
	Central retinal artery occlusion (CRAO)	(124, 140)
	Conjunctival chemosis	(136, 174–178)
	Conjunctival congestion	(172)
	Conjunctival hyperemia	(177–179)
	Conjunctival injection	(175)
	Conjunctival ischemia	(175)
	Corneal abrasions	(179–182)
	Corneal decompensation	(172)
	Corneal edema	(172, 175, 181, 183–186)
	Cornea epithelial defect	(172)
	Corneal infiltration	(172, 175, 180)
	Corneal scarring	(172, 186)
	Descemet membrane fold	(174)
	Endophthalmitis	(184, 187)
	Episcleral hyperemia	(178)
	Eyelid edema	(175, 179)
	Glaucoma	(172)
	Keratoconjunctivitis	(188)
	Keratopathy	(180, 186)
	Optic disc hyperemia	(189)
	Optic neuritis	(190)
	Optic neuropathy	(185)
	Retinal striae	(189)
	Scleritis	(184)
	Striate keratopathy	(177)
	Subconjunctival hemorrhage	(176, 185, 187)
	Uveitis	(189, 191)
	Vitritis	(189)

Melittin, phospholipase A_2_, apamin, and hyaluronidase are the primary toxic components of *Apis mellifera* bee venom. These substances significantly contribute to the development of clinical complications following multiple bee stings (192). The cardiovascular system is particularly vulnerable, with ischemic events, including acute myocardial infarction and Kounis syndrome (allergy-induced acute coronary syndrome), being common sequelae. Other potential cardiac complications include takotsubo cardiomyopathy, atrial fibrillation, and cardiac damage (193, 194).

Hemorrhage can occur in various locations following a bee sting, including the digestive, nervous, and respiratory systems, potentially leading to gastrointestinal, subarachnoid, or pulmonary hemorrhage, respectively. Additionally, hematological complications such as ischemia, anemia, thrombosis, hemolysis, disseminated intravascular coagulation (DIC), and shock, often culminating in hypovolemia, may arise. While hemolysis occurs in 17-22% of wasp sting cases, DIC is less common but can trigger thromboplastin release and microthrombi formation (192, 194).

Bee stings can induce hepatitis due to liver damage, manifested by elevated transaminases, alkaline phosphatases, and bilirubin levels. However, rhabdomyolysis and cardiac damage can also increase transaminases, complicating liver injury diagnosis. Severe liver damage may progress to liver failure. In rats, melittin has been implicated in liver injury through vasoconstriction and glycogenolysis (194).

Anaphylactic shock, an IgE-mediated immune response causing hypoperfusion and vasodilation, poses a severe risk to individuals with previous bee sting exposure or allergies. This life-threatening condition can lead to organ injury and death. Additionally, mast cell activation syndrome, characterized by excessive mast cell production and inflammatory effects, can trigger multisystem complications, organ failure, and mortality rates exceeding those of anaphylaxis (194).

Rhabdomyolysis, characterized by skeletal muscle breakdown, is frequently associated with bee envenoming, evidenced by elevated creatine phosphokinase (CPK) and bilirubin levels, and can contribute to acute kidney injury (AKI) (18, 192). Hemiparesis, or muscle weakness following ischemic stroke, is another potential muscular complication leading to immobilization or reduced physical activity (195).

Bee sting envenomation can lead to various neurological complications, including stroke (both ischemic and hemorrhagic), behavioral changes, ataxia, areflexia, encephalomyelitis, and Guillain-Barré syndrome. Ischemic and hemorrhagic strokes pose significant risks due to their potentially fatal outcomes. Subarachnoid intracranial hemorrhage often complicates ischemic stroke, undergoing hemorrhagic transformation. While the exact mechanisms remain unclear, two primary theories have been proposed: 1) an immune-mediated systemic reaction causing vasoconstriction and a prothrombotic state leading to ischemia and subsequent stroke; and 2) disseminated intravascular coagulation (DIC) triggered by tissue thromboplastin release, coupled with hemolysis, resulting in vessel occlusion, ischemic stroke, and eventual hemorrhagic transformation (9, 34).

Furthermore, behavioral changes, ataxia, and areflexia typically accompany degeneration or obstruction in specific regions of the brain and cerebellum. In this context, some cases displaying these complications have also experienced strokes, indicating a potential connection (196). It is well-established that the execution of any movement entails the coordinated action of agonist and synergist muscles, which contract to facilitate the movement, while antagonist muscles relax to allow it, and fixator muscles stabilize posture and prevent unintended shifts (196). Hence, considering that bee venom, particularly melittin, induces rhabdomyolysis, it is plausible that the partial loss of motor function in these cases may also be linked to rhabdomyolysis (18, 192).

Rhabdomyolysis, hemolysis, and hypotension can adversely affect the renal system, elevating creatine kinase and bilirubin levels, and ultimately leading to acute kidney injury (AKI) and renal failure. Multiple bee sting envenomation often causes glomerular and peritubular vasoconstriction, potentially resulting in ischemic injury and acute tubular necrosis. The accumulation of myoglobin, Tamm-Horsfall proteins, and uric acid within the tubules contributes to cast formation, further obstructing tubules and inducing ischemia. Anuria and hematuria are additional associated renal complications (192).

Clinical respiratory complications include acute pulmonary edema, pulmonary hemorrhage, and acute respiratory distress syndrome (ARDS). Bee venom’s ability to increase vascular permeability contributes to edema formation in various tissues, including the lungs, trachea, conjunctiva, and subcutaneous areas (194). Alveolar capillary damage can precipitate diffuse alveolar hemorrhage (DAH), potentially progressing to ARDS (197, 198). The pathogenesis of ARDS in bee sting envenomation remains unclear, although melittin and phospholipase A2 are suspected contributors to acute lung injury. Venom-induced inflammation can cause extensive tissue damage, culminating in severe cases of ARDS and multisystemic cardiorespiratory arrest (199, 200).

Ocular complications, though uncommon, encompass a wide range of issues. These include eyelid edema, conjunctival congestion, corneal abrasions, optic neuropathy, ptosis, purulent ocular secretions, conjunctival and episcleral hyperemia, symblepharon, macular retinal striae, ciliary congestion, corneal edema with Descemet’s membrane folding, and even vision loss. Additionally, lens abscess, partial iris atrophy, and cataract formation have been reported. The retained stinger’s direct venom action, coupled with the rapid immune response, primarily causes corneal injury (201, 202).

Clinical complications from bee sting envenomation can be severe, life-threatening, and multi-systemic. These complications, potentially triggered by a single sting, allergies, or anaphylaxis, can impact various bodily systems. Moreover, a high number of bee stings can exacerbate these complications, affecting the cardiovascular, nervous, hematological, or respiratory systems (**Table 2**).

**Table 2:** Clinical complications associated with honeybee stings envenoming in the entire patient cohort and in patients who died.

Case	Age	Sex	Country	Number of Stings	Time to Treatment	Time to Death	Comorbidity	Clinical complications	System	Reference
**1**	10	Male	Turkey	5989	3 h	12 days	–	Acute kidney failure Acute respiratory distress syndrome (ARDS) Convulsion Hemolysis Hypertension Ischemic stroke Multiorgan failure	Nervous Hematologic Respiratory Urinary Multiorgan	(134)
**2**	8	Male	Nigeria	–	50 min	36 h	Sickle cell anemia	Hematuria Hemolysis	Hematologic	(130)
**3**	25	Male	Australia	1	–	40 h	–	Acute respiratory distress syndrome (ARDS) Anaphylactic shock Bleeding Cerebral tonsillar herniation Disseminated intravascular coagulation (DIC) Hematuria Ischemic hepatitis Myocardial damage	Cardiovascular Hepatic Hematologic Immune Nervous Urinary Respiratory	(133)
**4**	38	Male	South Africa	32	–	–	–	Subarachnoid hemorrhage Subendocardial hemorrhage	Cardiovascular Nervous	(121)
**5**	58	Male	South Africa	1	–	–	–	Laryngeal edema Left ventricular hypertrophy Pulmonary congestion Pulmonary edema	Cardiovascular Respiratory	(121)
**6**	58	Male	South Africa	10	No medical care	–	–	Anaphylactic shock	Immune	(121)
**7**	38	Female	Hungary	1	No medical care	4 min	–	Acute respiratory distress syndrome (ARDS) Anaphylactic shock	Immune Respiratory	(73)
**8**	25	Male	Australia	1	–		Asthma	Cardiorespiratory arrest Laryngeal edema Pulmonary congestion	Cardiovascular Respiratory	(139)
**9**	36	Female	Australia	40-50	15 min	>29 min	Asthma Psoriasis	Cardiorespiratory arrest Laryngeal edema	Cardiovascular Respiratory	(139)
**10**	54	Male	Australia	1	15 min	>15 min	Osteoporosis	Cardiorespiratory arrest Laryngeal edema Pulmonary congestion Pulmonary edema	Cardiovascular Respiratory	(139)
**11**	70	Male	Brazil	–	20 h	–	Diabetes Hypertension	Acute respiratory distress syndrome (ARDS) Anuria Cardiorespiratory arrest	Cardiovascular Respiratory Urinary	(18)
**12**	39	Male	Honduras	–	3 h	37 h	–	Anaphylactic shock Anuria Rhabdomyolysis	Immune Multiorgan Muscular Urinary	(145)
**13**	19	Male	Brazil	2000	0 h	23 days	–	Acute respiratory distress syndrome (ARDS) Anuria Cardiorespiratory arrest Pericardial effusion Pulmonary edema Shock	Cardiovascular Hematologic Urinary Respiratory	(125)
**14**	46	Female	Brazil	1	2 h	2 h	Obesity	Anaphylactic shock Bleeding Subarachnoid hemorrhage Pulmonary edema Pulmonary hemorrhage	Hematologic Immune	(4)
**15**	48	Male	Turkey	–	–	3 days	–	Encephalitis Hepatitis Gastrointestinal haemorrhage	Digestory Hepatic Nervous	(128)
**16**	59	Male	Italy	–	–	–	–	Bronchial obstruction Cardiorespiratory arrest Laryngeal edema Pulmonary congestion Pulmonary edema Pulmonary emphysema Pulmonary hemorrhage	Cardiovascular Respiratory	(141)
**17**	67	Male	Iran	20	3 days	5 days	Type-2 diabetes Hypertension Myocardial infarction Ventricular aneurysm	Cardiorespiratory arrest Thrombotic thrombocytopenic purpura (TTP)	Cardiovascular Hematologic Respiratory	(137)
**18**	59	Male	India	–	–	5 days	Parkinson disease	Adrenal hemorrhage Laryngeal congestion Laryngeal edema Tracheal congestion Tracheal edema Pulmonary congestion Pulmonary edema Rhabdomyolysis	Muscular Urinary Respiratory	(138)
**19**	42	Male	Iran	–	–	30 min – 1 h	–	Anaphylactic shock Cardiorespiratory arrest	Cardiovascular Immune Respiratory	(143)
**20**	41	Female	India	1	3 h	7 days	–	Disseminated intravascular coagulation (DIC) Hemiparesis Hemorrhagic/Ischemic stroke	Hematologic Nervous	(149)
**21**	66	Male	Argentina	>500	–	36 h	Hypertension	Anaphylactic shock Multiorgan failure	Immune Multiorgan	(203)

Defining envenomation by sting number remains challenging, as the lethal venom dose is 2.8-3.5 mg/kg body weight (26, 28). Severe complications like disseminated intravascular coagulation, hematuria, ischemic hepatitis, myocardial damage, and death have been reported in adults with fewer than 30, even as few as one sting, particularly in sensitized individuals (**Table 2**). This highlights the unpredictable nature of bee sting envenomation.

Differentiating the causes and assessing the risks of death linked to either the direct toxic effects of venom or the allergic syndrome triggered by honeybee stings poses a formidable challenge, demanding thorough investigation and vigilant patient monitoring. Consequently, all incidents involving bee stings should be regarded as envenoming, and their significance, even in instances involving only a few stings, should not be underestimated. This recognition was underscored during the Apilic antivenom’s phase I/II clinical trial, wherein a recommendation was made to administer two vials of antivenom to patients with more than five stings. For those with fewer than five stings, the use of apilic antivenom is not advised, except when dictated by medical judgment (5).

## 6 Prevention of bee stings

Preventing severe systemic reactions to bee stings requires a multi-faceted approach. Clothing choices, such as wearing light-colored, smooth-textured garments, can deter bees. Avoiding scented products and maintaining personal hygiene by wearing clean clothes and showering regularly can also reduce the risk of stings. Minimizing exposure to bee-attracting factors, including flowering plants and food remnants, is crucial (204).

When confronted by a solitary stinging insect, remaining calm and still is crucial to avoid provocation. However, if multiple insects attack, swiftly retreating indoors or to a sheltered area is essential. Submerging in water to escape is ill-advised, as some bee species, like Africanized honeybees, may continue to sting upon resurfacing (205).

Individuals with a history of severe allergic reactions to insect stings should carry an epinephrine auto-injector and wear medical identification. Prompt medical attention is crucial in case of an allergic reaction (206). Integrating these preventive measures into daily routines significantly reduces the risk of severe systemic reactions from bee stings, enhancing safety in various environments.

## 7 Clinical complications diagnostic

Preventive exams identify diseases in asymptomatic individuals. Bee sting envenomations often present with multiple, late-diagnosed complications. Early diagnosis is crucial for effective management and improved outcomes (**Supplementary Table 1**). Although warning signs exist, monitoring challenges persist for general clinicians (207).

## 8 Pharmacological interventions

Current management and treatment protocols for honeybee sting envenomation primarily address analgesia and allergic reactions, with limited guidance on other complications. Consequently, treatment options and associated risks for these complications remain scarce. Given the diverse clinical manifestations of honeybee sting envenomation, adapting treatment protocols from other conditions may be beneficial. It is essential to consider both potential benefits and risks when implementing treatment plans, as some interventions may exacerbate complications or cause adverse effects. A comprehensive understanding of treatment options and their associated risks for various clinical complications is crucial for optimal patient care (**Supplementary Table 2**).

Symptomatic treatments for severe bee sting envenomations often prove ineffective. To address this, the Center for the Study of Venoms and Venomous Animals, Brazil initiated the development of a bee sting antivenom. Initial efforts focused on venom biochemical characterization to understand its composition. Traditional horse-based antivenom production faced challenges due to high allergic reaction rates and anaphylactic shock in horses. To mitigate these risks, researchers explored various strategies, including cobalt-60 irradiation (208). Irradiation significantly altered the chromatographic profiles of native Apis venom. While irradiated venom combined with IFA or SBA-15 produced similar antibody titers compared to native venom and IFA, these titers were notably lower. Importantly, irradiation reduced venom toxicity while preserving its immunogenicity, and IFA enhanced antibody production (208). However, successful immunization required removing all allergenic components from the venom, leaving only melittin and PLA2 for immunization.

Apilic antivenom demonstrated partial neutralization of venom effects, including hematocrit, vascular permeability, myeloperoxidase activity, edema, plasma CK activity, venom phospholipase and hyaluronidase activity, and cytotoxicity in kidney cell cultures (209, 210). These findings indicate the antivenom’s potential efficacy against Africanized bee (*A. mellifera*) venom and melittin *in vivo* and *in vitro*. Subsequent phase I/II clinical trials were initiated to evaluate the antivenom’s safety and efficacy. Given the absence of established clinical protocols for antivenom evaluation, a new protocol was developed and approved by ethics regulatory (The Brazilian Committee of Ethics in Research – CONEP) and sanitation agencies (Brazilian Health Regulatory Agency – ANVISA). This protocol outlined specific, adjuvant, symptomatic, and complementary treatments, in addition to standard clinical trial guidelines for heterologous antivenoms. It represented the first clinical trial specifically designed to assess the efficacy and safety of an Africanized bee venom antivenom (5).

The Apilic antivenom proved to be safe and demonstrated efficacy in neutralizing various venom effects, including hematocrit, vascular permeability, and enzyme activity, both *in vivo* and *in vitro*. These findings highlight the antivenom’s potential for treating Africanized bee (*A. mellifera*) envenomation. At the time, the pharmacokinetics of Africanized bee venom in humans was reported for the first time. The concentrations of melittin and PLA_2_ varied between 0.03 ng/mL and 587.35 ng/mL during hospitalization and follow-up, and interestingly, it was possible to observe that the blood concentration of PLA_2_ and mainly melittin increases again, especially after 10 days hospitalization, but without any clinical symptoms (8)). Mass spectrometry assays, allowed to determine the presence and relative levels of melittin in the participants. It should be noted that these issues were expected, as accidents involving AHB are peculiar and different from all other accidents involving venomous animals described. A phase III clinical trial is essential to confirm these observations, optimize dosing, and fully evaluate the antivenom’s efficacy (8).

The Apilic antivenom production process aligns with established antivenom manufacturing standards (211). However, producing pilot batches compliant with Good Manufacturing Practices (GMP) has presented challenges. Small-scale production for clinical trials is particularly difficult for pharmaceutical industries due to the associated risks and investments. Collaborating with Contract Development and Manufacturing Organizations (CDMOs) could facilitate technology transfer and small-batch production (212).

## 9 Final remarks

The increasing incidence of bee sting envenomation necessitates a comprehensive understanding of its underlying mechanisms, clinical manifestations, and potential complications to inform effective public health interventions. This review elucidates the intricate effects of bee venom and its associated health consequences. While existing research provides valuable insights into venom composition and its impact, clinical management guidelines for bee sting envenomation remain limited. This review fills a critical knowledge gap by providing a comprehensive overview of the clinical implications of bee venom exposure. Our analysis underscores the complexity of venom-human interactions and the severity of potential outcomes. The development of novel treatment strategies, including an antivenom, is imperative to reduce mortality and long-term complications. However, until such interventions become available, alternative pharmacological approaches must be explored, considering their associated risks.
