# Supplementary material for: Clinical complications in envenoming by Apis honeybee stings: insights into mechanisms, diagnosis, and pharmacological interventions
Source: Front Immunol. 2024 Sep 18;15:1437413. doi: 10.3389/fimmu.2024.1437413 (PMC11445026; doi:10.3389/fimmu.2024.1437413)
Supplement: Supplementary file 1 [file DataSheet1.pdf]

Supplementary Material

1 Supplementary Data

In instances of honeybee sting envenomation, patients frequently exhibit numerous clinical complications, often diagnosed belatedly. Timely identification of these complications can enhance their management and treatment. The Supplementary Material can be used as a guide to assist healthcare professionals in the treatment and management of symptoms in patients who are victims of this type of envenomation.

Table 1. Common complications associated with envenoming by massive honeybee stings, alert signals, and monitoring.

Complications	When to be alert	Monitoring	Ref.
Cerebral venous thrombosis (CVT)	Migraine and raised intracranial pressure (ICP)	Magnetic resonance imaging (MRI) and computed tomography (CT)	(1)
Subarachnoid Hemorrhage	Headache	Computed tomography (CT) and pay attention to erythrocytes	(2)
Acute coronary syndromes	Myocardial infarction and unstable angina	Cardiac troponin (cTn) measurements and change in electrocardiogram (ECG)	(3)
Kounis syndrome	Chest pain, palpitations, shortness of breath, rashes, hives and wheezing	Eosinophils counts, cardiac enzymes (CK, CK-MB) and troponin I or T, C-reactive protein, total and specific immunoglobulin E (IgE) Histamine, chymase, serum tryptase levels and arachidonic acid products.	(4)
Left ventricular hypertrophy	Extreme tiredness, dyspnea, chest pain, feeling of rapid heartbeat and fainting	Echocardiography, cardiovascular magnetic resonance imaging (MRI) and scintigraphy	(5)
Right Ventricular Failure	Chronic insufficiency, pulmonary embolism, chronic lung disease, pulmonary arterial	Attention to clinical signs, electrocardiogram (ECG) and imaging techniques.	(6)

	hypertension, and previous cardiomyopathies		
Atrioventricular Block	Severe cardiac arrhythmia, tiredness, nausea, and fainting	Monitoring the electrocardiogram (ECG)	(7)
Pericardial effusion	Dyspnea, left chest pain, feeling of chest fullness, swelling in the legs or abdomen	Chest X-ray, electrocardiogram, transthoracic echocardiography, computed tomography scan, cardiac magnetic resonance imaging, and pericardiocentesis.	(8)
Acute pericarditis	Acute Chest Pain	Attention to pericarditic chest pain, pericardial rub, saddle-shaped ST-elevation and/or PR-depression e non-trivial new or worsening pericardial effusion.	(9)
Myocardial infarction in the absence of obstructive coronary artery disease	Angina, extreme dyspnea during exertion, edema in the feet and pain in the shoulder or arm.	Invasive coronary angiography, multivessel intracoronary imaging, coronary spasm provocation test, and magnetic resonance imaging (MRI).	(10)
Takotsubo Syndrome	Dyspnea, precordialgia, orthopnea and/or acute pulmonary oedema or syncope	Electrocardiography, troponin I, troponin T, cardiac enzymes (CK-MB and troponin), and cardiac magnetic resonance	(11)
Boerhaave's syndrome	Vomiting, lower thoracic pain, and subcutaneous emphysema	Chest radiology examination	(12)
Upper gastrointestinal bleeding	Hematemesis and previous peptic ulcer	Early upper endoscopy, epinephrine injection, thermocoagulation, clipping and bandaging	(13)
Lower gastrointestinal bleeding	Blood in the anus or in the stool, abdominal pain, dizziness, increased heart rate, weakness, confusion, and collapse	Computed tomography (CT) abdomen or CT angiogram (0,5 mL/min), catheter angiography (detect bleeding at rates of 0.5 to 1.5 mL/min) and radionuclide imaging (0.1 mL/min)	(14,15)

Acute Limb Ischemia	Pain, paresthesia, muscle weakness, paralysis and even gangrene.	Use of Duplex ultrasound (DUS), Computed Tomography Angiography (CTA) and Magnetic Resonance Angiography (MRA) and Invasive Angiogram	(16)
Venous Thromboembolism	Previous deep vein thrombosis (DVT) and pulmonary embolism (PE)	Use thrombolytic therapy, prevention and use of anticoagulants	(17)
Disseminated Intravascular Coagulation (DIC)	Epistaxis, gingival bleeding, hematuria, oliguria, cough, dyspnea, fever, delirium, and coma	Thrombocytopenia, an elevated partial thromboplastin time, an elevated prothrombin time, increased levels of plasma D-dimers (or serum fibrin degradation products), and a decreasing plasma fibrinogen level	(18)
Hematochezia	Diverticulosis, angiodysplasia, neoplasm, perianal disorders, Meckel's diverticulum, colitis intussusception and others	Colonoscopy and arteriography	(19)
Thrombotic Thrombocytopenic Purpura	Fever, renal failure, hemolytic anemia, thrombocytopenia, and neurological changes	Use of the ADAMTS13 test in practice clinical	(20)
Acute Liver Failure	Fatigue/malaise, lethargy, anorexia, nausea and/or vomiting, pain in the right upper quadrant, pruritus, jaundice, and abdominal distension due to ascites	Monitoring liver markers (alanine transaminase [ALT], aspartate transaminase [AST], total bilirubin (TBL), and alkaline phosphatase (Alk phos) and underlying diseases	(21,22)
Anaphylaxis	Hypotension, tachycardia, urticaria, angioedema, wheezing, stridor, cyanosis, and syncope	Only clinical based on manifestations	(23)
Cardiac arrest	Absence of pulse and breathing	Sequence of thirty chest compressions for two ventilations	(24)
Rhabdomyolysis	Post-trauma patients, acute muscle weakness,	Monitoring laboratory tests and physical examination: elevated serum	(25)

	pain/tenderness and swelling (pain, tumor) of the affected extremity or region of the body	concentrations of CK (>5× the upper limit of normal or >1000 IU/L), myoglobin, lactate dehydrogenase (LDH), potassium, creatinine, and aspartate aminotransferase (AST)	
Acute Cerebellar Infarction	Lethargy, high blood pressure, vomiting	Continuous physical examination and use of tests such as magnetic resonance imaging (MRI) and computed tomography (CT)	(26)
Polyneuropathy	Tingling sensation, loss of sensitivity, pain, and loss of sense of vibration and position	Neurologic valuation	(27)
Guillain–Barré Syndrome	Drowsiness, mental confusion, coma, epileptic seizure, change in level of consciousness, loss of muscle coordination, double vision, facial and muscle weakness	Continuous monitoring of respiratory function, Intravenous immunoglobulin	(28)
Encephalitis	Fever, Headache, Personality changes or confusion.<br><br>Seizures, Paralysis or lack of sensation, Drowsiness	Magnetic resonance imaging (MRI) and computed tomography (CT) and reassessment of viral, bacterial, fungal, protozoal, and helminthic etiologies	(29)
Spontaneous Intracerebral Hemorrhage	Headache, Loss of consciousness, Bodily weakness or lethargy, Sudden epileptic seizures, Occurrence of nausea and vomiting and Numbness or tingling.	Magnetic resonance imaging (MRI) and computed tomography (CT)	(30)
Acute Ischemic Stroke	Dyslalia, Paralysis or numbness in the face, arm or leg, on only one side of the body, Sudden and intense headache, Dizziness that may be accompanied by	Magnetic resonance imaging (MRI) and computed tomography (CT)	(31)

	vomiting, Loss of motor coordination		
Acute Intracerebral Haemorrhage	Migraine, Loss of consciousness, Bodily weakness or lethargy, Occurrence of nausea and vomiting, Numbness or tingling, Motor problems and loss of coordination	Magnetic resonance imaging (MRI) and computed tomography (CT) for support	(32)
Aneurysmal Subarachnoid Hemorrhage	Suspected rupture of an aneurysm	Magnetic resonance imaging (MRI) and computed tomography (CT) for support	(33)
Chronic Subdural Hematoma	Migraine, drowsiness, confusion, memory changes, paralysis on the side of the body opposite the hematoma and dyslalia	Follow-up with imaging exams such as magnetic resonance imaging (MRI) and computed tomography (CT)	(34)
Acute Kidney Injury (AKI)	Decreased urine production, Fluid retention, causing edema in the legs, ankles or feet, Drowsiness, Lack of appetite, dyspnea, fatigue, confusion, severe hyperkalemia, and others	Increase in serum creatinine by ≥0.3 mg/dl (≥26.5 μmol/l) in 48 hours; OR<br>Increase in serum creatinine to ≥1.5 times the baseline value, which is known or presumed to have occurred within the last 7 days: OR Urine volume <0.5 ml/kg/h for 6 hours	(35)
Obstructive Pulmonary	Dyspnea on exertion, throat clearing, forced expiratory time (FET) of more than six seconds is suggestive of airflow obstruction, arterial blood gas analysis should be done if arterial saturation by pulse oximetry is less than 90%	Spirometry, lung (pulmonary) function tests, chest X-ray, CT scan, arterial blood gas analysis	(36)
Acute Respiratory Distress Syndrome	Sudden onset fever, chills, headache, cough, runny nose, sore throat, difficulty smelling or tasting, dyspnea	Ventilatory support	(37)

Acute Pulmonary Edema	Intense dyspnea, restlessness, and anxiety, as well as a feeling of suffocation	Chest X-ray, chest computerized tomography (CT) scan, pulse oximetry, arterial blood gas test, and ultrasound of the lungs	(38)

Table 2. Risks of common drugs and others conducts used to treat patients with envenoming by massive honeybee stings.

Clinical Complication	Drug/ Treatment	Risk	Reference
Acute kidney failure	Fluid resuscitation with isotonic crystalloid, correction of acid-base disorders, hydroelectrolytes, diuretics, hemodialysis	Errors in prescription may lead to significant complications	(39)
Acute respiratory distress syndrome (ARDS)	Bonsetan	hemolytic anemia	(40,41)
	Mechanical ventilation	Lung injury	(42)
Convulsion	Benzodiazepines	Decreased psychomotor activity and respiratory	(43)
Hemolysis	Corticosteroids	Decrease bone density	(44,45)
Ischemic stroke	Thrombolytic therapy	Intracerebral hemorrhage, Angioedema, Surgery may be required	(46) (47)
Hematuria	Hydration	-	(48)
Anaphylactic shock	Epinephrine	Digital block (gangrene)	(49,50)
Bleeding	Antifibrinolytic drugs	-	(51)
Cerebral tonsillar herniation	Drainage	-	(52,53)

Disseminated intravascular coagulation (DIC)	Heparin	Thrombocytopenia	(54,55)
Ischemic hepatitis	Inotropes associated with diuretics	Arrhythmia, cardiac micronecrosis, and death	(56)
Myocardial damage	Streptokinase	Cerebrovascular complications	(57,58)
Subarachnoid hemorrhage	Surgical or endovascular treatment	Death	(59)
Laryngeal and Tracheal edema/ congestion	Steroids	Digital block (gangrene)	(60,61)
Left ventricular hypertrophy	Statins	Muscle complications	(62,63)
Pulmonary congestion	Mechanical ventilation	Lung injury	(42,64)
Pulmonary edema	Diuretic and Hyperosmotic colloidal solution	Kidney complications	(65,66)
Cardiorespiratory arrest	Defibrillation, chest compressions, use of advanced airways, extracorporeal cardiopulmonary, resuscitation, vasopressor therapy	Elevated heart rate, tachyarrhythmia, myocardial ischemia, cell damage	(67,68)
Rhabdomyolysis	Fluid replacement therapy	Hypervolemia, hypertension and hydroelectrolyte disorders	(69)
Anuria	Thrombectomy, and dialysis	Ischemic damage	(70,71)

Pericardial effusion	Pericardiocentesis, Pericardial window, Pericardial catheter drainage or Sclerosing therapies	Ventricular tachycardia, Injury to intercostal vessels, chamber laceration, pneumothorax, effusive, constrictive pericarditis	(72)
Encephalitis	Corticoestroids	Decrease bone density	(44,73)
Hepatitis	n-Acetylcysteine	Flushing, rash, pruritus, bronchospasms	(74,75)
Gastrointestinal hemorrhage	Epinephrine	Digital block (gangrene)	(49,76)
Bronchial obstruction	Corticosteroids	Decrease bone density	(44,77)
Pulmonary emphysema	Bronchodilators	Increased heart rate, palpitations, tachyarrhythmia	(78)
Pulmonary hemorrhage	Tranexamic acid	Neurological events (only when administered in high dose)	(79)
Thrombotic thrombocytopenic purpura (TTP)	Plasma-exchange	Paresthesias, hematoma, clotting, allergic reactions, bleeding	(80)
Adrenal hemorrhage	Surgical Treatment	Death	(81)

Hemiparesis	Physiotherapy, Electrical stimulation	-	(82)

References

1. Behrouzi R, Punter M. Diagnosis and management of cerebral venous thrombosis. *Clinical Medicine* (2018) 18:75–79. doi: 10.7861/clinmedicine.18-1-75

2. Marcolini E, Hine J. Approach to the Diagnosis and Management of Subarachnoid Hemorrhage. *Western Journal of Emergency Medicine* (2019) 20:203–211. doi: 10.5811/westjem.2019.1.37352

3. Eng-Frost J, Chew C. Diagnosis and management of acute coronary syndromes. *Aust Prescr* (2021) 44:180–184. doi: 10.18773/austprescr.2021.049

4. Abdelghany M, Subedi R, Shah S, Kozman H. Kounis syndrome: A review article on epidemiology, diagnostic findings, management and complications of allergic acute coronary syndrome. *Int J Cardiol* (2017) 232:1–4. doi: 10.1016/j.ijcard.2017.01.124

5. Moura B, Aimo A, Al-Mohammad A, Keramida K, Ben Gal T, Dorbala S, Todiere G, Cameli M, Barison A, Bayes-Genis A, et al. Diagnosis and management of patients with left ventricular hypertrophy: Role of multimodality cardiac imaging. A scientific statement of the Heart Failure Association of the European Society of Cardiology. *Eur J Heart Fail* (2023) doi: 10.1002/ejhf.2997

6. Arrigo M, Huber LC, Winnik S, Mikulicic F, Guidetti F, Frank M, Flammer AJ, Ruschitzka F. Right Ventricular Failure: Pathophysiology, Diagnosis and Treatment. *Card Fail Rev* (2019) 5:140–146. doi: 10.15420/cfr.2019.15.2

7. Kashou AH, Goyal A, Nguyen T, Ahmed I, Chhabra L. *Atrioventricular Block*. (2023). 1 p.

8. Yamani N, Abbasi A, Almas T, Mookadam F, Unzek S. Diagnosis, treatment, and management of pericardial effusion- review. *Annals of Medicine & Surgery* (2022) 80: doi: 10.1016/j.amsu.2022.104142

9. Ismail TF. Acute pericarditis: Update on diagnosis and management. *Clinical Medicine* (2020) 20:48–51. doi: 10.7861/clinmed.cme.20.1.4

10. Tamis-Holland JE, Jneid H, Reynolds HR, Agewall S, Brilakis ES, Brown TM, Lerman A, Cushman M, Kumbhani DJ, Arslanian-Engoren C, et al. Contemporary Diagnosis and Management of Patients With Myocardial Infarction in the Absence of Obstructive Coronary Artery Disease: A Scientific Statement From the American Heart Association. *Circulation* (2019) 139: doi: 10.1161/CIR.0000000000000670

11. Assad J, Femia G, Pender P, Badie T, Rajaratnam R. Takotsubo Syndrome: A Review of Presentation, Diagnosis and Management. *Clin Med Insights Cardiol* (2022) 16:117954682110657. doi: 10.1177/11795468211065782

12. Wang J, Wang D, Chen J. Diagnostic challenge and surgical management of Boerhaave's syndrome: a case series. *J Med Case Rep* (2021) 15:553. doi: 10.1186/s13256-021-03080-1

13. Wilkins T, Khan N, Nabh A, Schade RR. Diagnosis and management of upper gastrointestinal bleeding. *Am Fam Physician* (2012) 85:469–76.

14. Triantafyllou K, Gkolfakis P, Gralnek IM, Oakland K, Manes G, Radaelli F, Awadie H, Camus Duboc M, Christodoulou D, Fedorov E, et al. Diagnosis and management of acute lower gastrointestinal bleeding: European Society of Gastrointestinal Endoscopy (ESGE) Guideline. *Endoscopy* (2021) 53:850–868. doi: 10.1055/a-1496-8969

15. Kim BSM. Diagnosis of gastrointestinal bleeding: A practical guide for clinicians. *World J Gastrointest Pathophysiol* (2014) 5:467. doi: 10.4291/wjgp.v5.i4.467

16. Olinic D-M, Stanek A, Tătaru D-A, Homorodean C, Olinic M. Acute Limb Ischemia: An Update on Diagnosis and Management. *J Clin Med* (2019) 8:1215. doi: 10.3390/jcm8081215

17. Ortel TL, Neumann I, Ageno W, Beyth R, Clark NP, Cuker A, Hutten BA, Jaff MR, Manja V, Schulman S, et al. American Society of Hematology 2020 guidelines for management of venous thromboembolism: treatment of deep vein thrombosis and pulmonary embolism. *Blood Adv* (2020) 4:4693–4738. doi: 10.1182/bloodadvances.2020001830

18. Levi M, Scully M. How I treat disseminated intravascular coagulation. *Blood* (2018) 131:845–854. doi: 10.1182/blood-2017-10-804096

19. Wandono H. Diagnosis and treatment of hematochezia: guideline for clinical practice. *Acta Med Indones* (2007) 39:202–6.

20. Zheng XL, Vesely SK, Cataland SR, Coppo P, Geldziler B, Iorio A, Matsumoto M, Mustafa RA, Pai M, Rock G, et al. ISTH guidelines for the diagnosis of thrombotic thrombocytopenic purpura. *Journal of Thrombosis and Haemostasis* (2020) 18:2486–2495. doi: 10.1111/jth.15006

21. Larson AM. Diagnosis and management of acute liver failure. *Curr Opin Gastroenterol* (2010) 26:214–221. doi: 10.1097/MOG.0b013e32833847c5

22. Punzalan CS, Barry CT. Acute Liver Failure. *J Intensive Care Med* (2016) 31:642–653. doi: 10.1177/0885066615609271

23. Navalpakam A, Thanaputkaiporn N, Poowuttikul P. Management of Anaphylaxis. *Immunol Allergy Clin North Am* (2022) 42:65–76. doi: 10.1016/j.iac.2021.09.005

24. Lott C, Truhlář A, Alfonzo A, Barelli A, González-Salvado V, Hinkelbein J, Nolan JP, Paal P, Perkins GD, Thies K-C, et al. European Resuscitation Council Guidelines 2021: Cardiac arrest in special circumstances. *Resuscitation* (2021) 161:152–219. doi: 10.1016/j.resuscitation.2021.02.011

25. Kodadek L, Carmichael SP, Seshadri A, Pathak A, Hoth J, Appelbaum R, Michetti CP, Gonzalez RP. Rhabdomyolysis: an American Association for the Surgery of Trauma Critical Care Committee Clinical Consensus Document. *Trauma Surg Acute Care Open* (2022) 7:e000836. doi: 10.1136/tsaco-2021-000836

26. Wright J, Huang C, Strbian D, Sundararajan S. Diagnosis and Management of Acute Cerebellar Infarction. *Stroke* (2014) 45:1–10. doi: 10.1161/STROKEAHA.114.004474

27. Mirian A, Aljohani Z, Grushka D, Florendo-Cumbermack A. Diagnosis and management of patients with polyneuropathy. *Can Med Assoc J* (2023) 195:E227–E233. doi: 10.1503/cmaj.220936

28. Leonhard SE, Mandarakas MR, Gondim FAA, Bateman K, Ferreira MLB, Cornblath DR, van Doorn PA, Dourado ME, Hughes RAC, Islam B, et al. Diagnosis and management of Guillain–Barré syndrome in ten steps. *Nat Rev Neurol* (2019) 15:671–683. doi: 10.1038/s41582-019-0250-9

29. Tunkel AR, Glaser CA, Bloch KC, Sejvar JJ, Marra CM, Roos KL, Hartman BJ, Kaplan SL, Scheld WM, Whitley RJ. The Management of Encephalitis: Clinical Practice Guidelines by the Infectious Diseases Society of America. *Clinical Infectious Diseases* (2008) 47:303–327. doi: 10.1086/589747

30. Greenberg SM, Ziai WC, Cordonnier C, Dowlatshahi D, Francis B, Goldstein JN, Hemphill JC, Johnson R, Keigher KM, Mack WJ, et al. 2022 Guideline for the Management of Patients With Spontaneous Intracerebral Hemorrhage: A Guideline From the American Heart Association/American Stroke Association. *Stroke* (2022) 53:1–10. doi: 10.1161/STR.0000000000000407

31. Powers WJ, Rabinstein AA, Ackerson T, Adeoye OM, Bambakidis NC, Becker K, Biller J, Brown M, Demaerschalk BM, Hoh B, et al. Guidelines for the Early Management of Patients With Acute Ischemic Stroke: 2019 Update to the 2018 Guidelines for the Early Management of Acute Ischemic Stroke: A Guideline for Healthcare Professionals From the American Heart Association/American Stroke. *Stroke* (2019) 50: doi: 10.1161/STR.0000000000000211

32. McGurgan IJ, Ziai WC, Werring DJ, Al-Shahi Salman R, Parry-Jones AR. Acute intracerebral haemorrhage: diagnosis and management. *Pract Neurol* (2021) 21:128–136. doi: 10.1136/practneurol-2020-002763

33. Hoh BL, Ko NU, Amin-Hanjani S, Hsiang-Yi Chou S, Cruz-Flores S, Dangayach NS, Derdeyn CP, Du R, Hänggi D, Hetts SW, et al. *2023 Guideline for the Management of Patients With Aneurysmal Subarachnoid Hemorrhage: A Guideline From the American Heart Association/American Stroke Association*. (2023). 314–370 p. doi: 10.1161/STR.0000000000000436

34. Mehta V, Harward SC, Sankey EW, Nayar G, Codd PJ. Evidence based diagnosis and management of chronic subdural hematoma: A review of the literature. *Journal of Clinical Neuroscience* (2018) 50:7–15. doi: 10.1016/j.jocn.2018.01.050

35. Okusa MD, Rosner MH. Overview of the management of acute kidney injury (AKI) in adults. *UpToDate* (2023) 1:1–10.

36. Gupta D, Agarwal R, Aggarwal A, Maturu V, Dhooria S, Prasad K, Sehgal I, Yenge L, Jindal A, Singh N, et al. Guidelines for diagnosis and management of chronic obstructive pulmonary disease: Joint ICS/NCCP (I) recommendations. *Lung India* (2013) 30:228. doi: 10.4103/0970-2113.116248

37. Griffiths MJD, McAuley DF, Perkins GD, Barrett N, Blackwood B, Boyle A, Chee N, Connolly B, Dark P, Finney S, et al. Guidelines on the management of acute respiratory distress syndrome. *BMJ Open Respir Res* (2019) 6:e000420. doi: 10.1136/bmjresp-2019-000420

38. Purvey M, Allen G. Managing acute pulmonary oedema. *Aust Prescr* (2017) 40:59–63. doi: 10.18773/austprescr.2017.013

39. Mercado MG, Smith DK, Guard EL. Acute kidney injury: Diagnosis and management. *Am Fam Physician* (2019) 100:687–694.

40. Afra F, Mehri M, Namazi S. Bosentan-induced immune hemolytic anemia in 17 years old man. A case report. *DARU Journal of Pharmaceutical Sciences* (2021) 29:211–215. doi: 10.1007/s40199-020-00386-1

41. Araz O. Current Pharmacological Approach to ARDS: The Place of Bosentan. *Eurasian J Med* (2020) 52:81–85. doi: 10.5152/eurasianjmed.2020.19218

42. Mahboobi SK, Sohail M. "Complications and Side Effects of Mechanical Ventilation.," In: Abd-Elsayed A, editor. *Advanced Anesthesia Review*. Oxford University PressNew York (2023). p. 277-C109.S15 doi: 10.1093/med/9780197584521.003.0108

43. Edinoff AN, Nix CA, Hollier J, Sagrera CE, Delacroix BM, Abubakar T, Cornett EM, Kaye AM, Kaye AD. Benzodiazepines: Uses, Dangers, and Clinical Considerations. *Neurol Int* (2021) 13:594–607. doi: 10.3390/neurolint13040059

44. Van Staa TP, Leufkens HGM, Abenhaim L, Zhang B, Cooper C. Use of Oral Corticosteroids and Risk of Fractures. *Journal of Bone and Mineral Research* (2000) 15:993–1000. doi: 10.1359/jbmr.2000.15.6.993

45. Barcellini W. Immune Hemolysis: Diagnosis and Treatment Recommendations. *Semin Hematol* (2015) 52:304–312. doi: 10.1053/j.seminhematol.2015.05.001

46. Phipps MS, Cronin CA. Management of acute ischemic stroke. *BMJ* (2020)l6983. doi: 10.1136/bmj.l6983

47. Lapchak PA, Araujo DM. Advances in hemorrhagic stroke therapy: conventional and novel approaches. *Expert Opin Emerg Drugs* (2007) 12:389–406. doi: 10.1517/14728214.12.3.389

48. Vedula R, Iyengar AA. Approach to Diagnosis and Management of Hematuria. *The Indian Journal of Pediatrics* (2020) 87:618–624. doi: 10.1007/s12098-020-03184-4

49. Denkler K. A Comprehensive Review of Epinephrine in the Finger: To Do or Not to Do. *Plast Reconstr Surg* (2001) 108:114–124. doi: 10.1097/00006534-200107000-00017

50. Barach EM. Epinephrine for Treatment of Anaphylactic Shock. *JAMA: The Journal of the American Medical Association* (1984) 251:2118. doi: 10.1001/jama.1984.03340400046024

51. Ker K, Roberts I, Shakur H, Coats TJ. Antifibrinolytic drugs for acute traumatic injury. *Cochrane Database of Systematic Reviews* (2015) doi: 10.1002/14651858.CD004896.pub4

52. Sugrue PA, Hsieh PC, Getch CC, Batjer HH. Acute symptomatic cerebellar tonsillar herniation following intraoperative lumbar drainage. *J Neurosurg* (2009) 110:800–803. doi: 10.3171/2008.5.17568

53. Lo WB, Thant KZ, Kaderbhai J, White N, Nishikawa H, Dover MS, Evans M, Rodrigues D. Posterior calvarial distraction for complex craniosynostosis and cerebellar tonsillar herniation. *J Neurosurg Pediatr* (2020) 26:421–430. doi: 10.3171/2020.4.PEDS19742

54. Arepally GM. Heparin-induced thrombocytopenia. *Blood* (2017) 129:2864–2872. doi: 10.1182/blood-2016-11-709873

55. Levi M, Scully M. How I treat disseminated intravascular coagulation. *Blood* (2018) 131:845–854. doi: 10.1182/blood-2017-10-804096

56. CIOBANU AO, GHERASIM L. Ischemic Hepatitis – Intercorrelated Pathology. *MAEDICA – a Journal of Clinical Medicine* (2018) 13:5–11. doi: 10.26574/maedica.2018.13.1.5

57. Aldrich MS. Cerebrovascular Complications of Streptokinase Infusion. *JAMA: The Journal of the American Medical Association* (1985) 253:1777. doi: 10.1001/jama.1985.03350360103029

58. Koren G, Weiss AT, Hasin Y, Appelbaum D, Welber S, Rozenman Y, Lotan C, Mosseri M, Sapoznikov D, Luria MH, et al. Prevention of Myocardial Damage in Acute Myocardial Ischemia by Early Treatment with Intravenous Streptokinase. *New England Journal of Medicine* (1985) 313:1384–1389. doi: 10.1056/NEJM198511283132204

59. Lawton MT, Vates GE. Subarachnoid Hemorrhage. *New England Journal of Medicine* (2017) 377:257–266. doi: 10.1056/NEJMcp1605827

60. Johnson L. Tracheal Collapse. *Veterinary Clinics of North America: Small Animal Practice* (2000) 30:1253–1266. doi: 10.1016/S0195-5616(00)06005-8

61. Pluijms WA, van Mook WN, Wittekamp BH, Bergmans DC. Postextubation laryngeal edema and stridor resulting in respiratory failure in critically ill adult patients: updated review. *Crit Care* (2015) 19:295. doi: 10.1186/s13054-015-1018-2

62. El-Salem K, Ababneh B, Rudnicki S, Malkawi A, Alrefai A, Khader Y, Saadeh R, Saydam M. Prevalence and risk factors of muscle complications secondary to statins. *Muscle Nerve* (2011) 44:877–881. doi: 10.1002/mus.22205

63. Simko F. Statins: a perspective for left ventricular hypertrophy treatment. *Eur J Clin Invest* (2007) 37:681–691. doi: 10.1111/j.1365-2362.2007.01837.x

64. Lüsebrink E, Orban M, Kupka D, Scherer C, Hagl C, Zimmer S, Luedike P, Thiele H, Westermann D, Massberg S, et al. Prevention and treatment of pulmonary congestion in patients undergoing venoarterial extracorporeal membrane oxygenation for cardiogenic shock. *Eur Heart J* (2020) 41:3753–3761. doi: 10.1093/eurheartj/ehaa547

65. Greenberg Arthur. Diuretic Complications. *Am J Med Sci* (2000) 319:10–24. doi: 10.1016/S0002-9629(15)40676-7

66. Rathi RK, Jindal S, Joseph HT, Bal S. Unilobar re-expansion pulmonary oedema following removal of a large intra-thoracic mass. *Indian J Thorac Cardiovasc Surg* (2023) 39:68–71. doi: 10.1007/s12055-022-01418-y

67. Holmberg MJ, Issa MS, Moskowitz A, Morley P, Welsford M, Neumar RW, Paiva EF, Coker A, Hansen CK, Andersen LW, et al. Vasopressors during adult cardiac arrest: A systematic review and meta-analysis. *Resuscitation* (2019) 139:106–121. doi: 10.1016/j.resuscitation.2019.04.008

68. Schmittinger CA, Torgersen C, Luckner G, Schröder DCH, Lorenz I, Dünser MW. Adverse cardiac events during catecholamine vasopressor therapy: a prospective observational study. *Intensive Care Med* (2012) 38:950–958. doi: 10.1007/s00134-012-2531-2

69. Cabral BMI, Edding SN, Portocarrero JP, Lerma E V. Rhabdomyolysis. *Disease-a-Month* (2020) 66:101015. doi: 10.1016/j.disamonth.2020.101015

70. FLYE MW, ANDERSON RW, FISH JC, SILVER D. Successful Surgical Treatment of Anuria Caused by Renal Artery Occlusion. *Ann Surg* (1982) 195:346–353. doi: 10.1097/00000658-198203000-00016

71. Kaesmacher J, Kaesmacher M, Maegerlein C, Zimmer C, Gersing AS, Wunderlich S, Friedrich B, Boeckh-Behrens T, Kleine JF. Hemorrhagic Transformations after Thrombectomy: Risk Factors and Clinical Relevance. *Cerebrovascular Diseases* (2017) 43:294–304. doi: 10.1159/000460265

72. Yamani N, Abbasi A, Almas T, Mookadam F, Unzek S. Diagnosis, treatment, and management of pericardial effusion- review. *Annals of Medicine & Surgery* (2022) 80: doi: 10.1016/j.amsu.2022.104142

73. Tripathy S, Routray PK, Mohapatra AK, Mohapatra M, Dash SC. Acute Demyelinating Encephalomyelitis After Anti-venom Therapy in Russell's Viper Bite. *Journal of Medical Toxicology* (2010) 6:318–321. doi: 10.1007/s13181-010-0015-8

74. Koppen A, Van Riel A, De Vries I, Meulenbelt J. Recommendations for the paracetamol treatment nomogram and side effects of N-acetylcysteine *corresponding author: a.koppen@umcutrecht.nl. (2014)251–257.

75. Wang D-W. Advances in the management of acute liver failure. *World J Gastroenterol* (2013) 19:7069. doi: 10.3748/wjg.v19.i41.7069

76. Raphaeli T, Menon R. Current Treatment of Lower Gastrointestinal Hemorrhage. *Clin Colon Rectal Surg* (2012) 25:219–227. doi: 10.1055/s-0032-1329393

77. Roisman GL, Peiffer C, Lacronique JG, Le Cae A, Dusser DJ. Perception of bronchial obstruction in asthmatic patients. Relationship with bronchial eosinophilic inflammation and epithelial damage and effect of corticosteroid treatment. *Journal of Clinical Investigation* (1995) 96:12–21. doi: 10.1172/JCI118011

78. Matera MG, Rogliani P, Calzetta L, Cazzola M. Safety Considerations with Dual Bronchodilator Therapy in COPD: An Update. *Drug Saf* (2016) 39:501–508. doi: 10.1007/s40264-016-0402-4

79. Hunt BJ. The current place of tranexamic acid in the management of bleeding. *Anaesthesia* (2015) 70:50. doi: 10.1111/anae.12910

80. Basic-Jukic N, Kes P, Glavas-Boras S, Brunetta B, Bubic-Filipi L, Puretic Z. Complications of Therapeutic Plasma Exchange: Experience With 4857 Treatments. *Therapeutic Apheresis and Dialysis* (2005) 9:391–395. doi: 10.1111/j.1744-9987.2005.00319.x

81. Karwacka I, Obolonczyk L, Sworczak K. Adrenal hemorrhage: A single center experience and literature review. *Advances in Clinical and Experimental Medicine* (2018) 27:681–687. doi: 10.17219/acem/68897

82. Urton ML, Kohia M, Davis J, Neill MR. Systematic literature review of treatment interventions for upper extremity hemiparesis following stroke. *Occup Ther Int* (2007) 14:11–27. doi: 10.1002/oti.220
